# Exploring the Potential of Zirconium-89 in Diagnostic Radiopharmaceutical Applications: An Analytical Investigation

**DOI:** 10.3390/biomedicines11041173

**Published:** 2023-04-13

**Authors:** Ahmed M. A. Mostafa, Hesham M. H. Zakaly, Shams A. M. Issa, Mohamed A. M. Uosif, Ziyad A. Alrowaili, Michael V. Zhukovsky

**Affiliations:** 1 Physics Department, College of Science, Jouf University, Sakaka P.O. Box 2014, Saudi Arabia; 2 Institute of Physics and Technology, Ural Federal University, Ekaterinburg 620002, Russia; 3 Physics Department, Faculty of Science, Al-Azhar University, Assiut Branch, Assiut 71524, Egypt; 4 Department of Physics, Faculty of Science, University of Tabuk, Tabuk P.O. Box 47512, Saudi Arabia; 5 Institute of Industrial Ecology UB RAS, Ekaterinburg 620137, Russia

**Keywords:** coefficients of transition, radiopharmaceuticals, exponential function, clinical data, biological distribution

## Abstract

This study highlights the use of ^89^Zr-oxalate in diagnostic applications with the help of WinAct and IDAC2.1 software. It presents the biodistribution of the drug in various organs and tissues, including bone, blood, muscle, liver, lung, spleen, kidneys, inflammations, and tumors, and analyzes the maximum amount of nuclear transformation per Bq intake for each organ. The retention time of the maximum nuclear transformation and the absorbed doses of the drug in various organs and tissues are also examined. Data from clinical and laboratory studies on radiopharmaceuticals are used to estimate the coefficients of transition. The accumulation and excretion of the radiopharmaceutical in the organs is assumed to follow an exponential law. The coefficients of transition from the organs to the blood and vice versa are estimated using a combination of statistical programs and digitized data from the literature. WinAct and IDAC 2.1 software are used to calculate the distribution of the radiopharmaceutical in the human body and to estimate the absorbed doses in organs and tissues. The results of this study can provide valuable information for the biokinetic modeling of wide-spectrum diagnostic radiopharmaceuticals. The results show that ^89^Zr-oxalate has a high affinity for bones and a relatively low impact on healthy organs, making it helpful in targeting bone metastases. This study provides valuable information for further research on the development of this drug for potential clinical applications.

## 1. Introduction

The use of radionuclides in medicine has been an important area of research and development for several decades. One such radionuclide that has gained significant interest in recent years is ^89^Zr, owing to its favorable physical properties, including a long half-life of 78.4 h and a high-energy positron emission that enables sensitive PET imaging [1,2,3,4,5]. While ^89^Zr has traditionally been used for labeling monoclonal antibodies (mAbs) in imaging, there has been growing interest in exploring other labeling strategies that could expand the clinical applications of this radionuclide. This interest stems from the fact that mAbs have become a critical component in targeted cancer therapy, owing to their ability to specifically bind to cancer cells and deliver cytotoxic agents or other therapeutic payloads. However, the success of these therapies depends on the accurate detection and monitoring of cancer cells, which can be challenging when using conventional imaging techniques. One such strategy is the use of ^89^Zr-oxalate for the labeling of small molecules, peptides, and other biomolecules [6,7].

^89^Zr-oxalate has several advantages over other labeling strategies, including ease of synthesis, high labeling efficiency, and stability in biological matrices. Moreover, the use of ^89^Zr-oxalate allows for the preparation of diagnostic radiopharmaceuticals with high specific activity, which can improve the sensitivity and accuracy of PET imaging and therapy [3,8].

The use of monoclonal antibodies (mAbs) labeled with radiometals for imaging and therapy has emerged as a promising approach in cancer diagnosis and treatment. The choice of radionuclide is critical for achieving high-quality images with high sensitivity and specificity, as well as for delivering therapeutic doses to the target tissue while minimizing radiation exposure to healthy tissues [9,10,11]. ^89^Zr, owing to its favorable physical properties, including a long half-life, good positron yield, and low radiation dose, is an ideal radionuclide for the labeling of mAbs and other biological molecules for PET imaging. The slow blood clearance of most IgGs used in radioimmunodiagnosis is compatible with the long half-life of ^89^Zr. The maximum accumulation of an IgG in the tumor is around 3–5 days due to the slow clearance of these molecules from the bloodstream. The long half-life of ^89^Zr allows for delayed imaging, which can improve the sensitivity and accuracy of PET imaging [12].

The conjugation of ^89^Zr to mAbs is typically achieved via a deferoxamine (DFO) moiety, which can chelate the ^89^Zr ion with high affinity and stability [13]. This conjugation strategy has been widely used in preclinical and clinical studies, demonstrating high target-to-background ratios and excellent imaging contrast for various cancer types [14,15].

Overall, the use of ^89^Zr-labeled mAbs for PET imaging is a rapidly evolving field with enormous potential to improve the diagnosis and treatment of cancer. The combination of the long half-life of ^89^Zr and slow blood clearance of most IgGs used in radioimmunodiagnosis offers a promising strategy for achieving high-quality images with high sensitivity and specificity. The ongoing development of new ^89^Zr-labeled mAbs and other biological molecules is expected to further expand the clinical applications of this technology in the future.

Zirconium is a naturally occurring element that has been found to accumulate preferentially in bone tissue in both rats [16,17] and mice [2,18]. This property has been attributed to zirconium’s chemical similarity to other bone-seeking elements, such as calcium and phosphorus. In recent years, significant research has been aimed at optimizing the use of ^89^Zr-oxalate for developing new radiopharmaceuticals for a range of applications, including cancer diagnosis, inflammation imaging, and cardiovascular imaging [19]. In this regard, it is of interest to evaluate the dose loads on human organs and tissues when using zirconium oxalate labeled with the nuclide ^89^Zr for the diagnosis and PET imaging of tumors and inflammations. Since there are no direct data on the use of this radiopharmaceutical in humans, these calculations should be carried out by extrapolation of data obtained from laboratory animals to humans. The novelty of this study lies in the evaluation of the biodistribution and dosimetry of ^89^Zr-oxalate, a radiopharmaceutical that has shown promise for use in PET imaging. This study provides new information on the coefficients of transition for ^89^Zr-oxalate, which are critical for accurately predicting the distribution and accumulation of the radiopharmaceutical in various organs and tissues. Additionally, the study presents new data on the absorbed doses of ^89^Zr-oxalate in different organs and tissues after injection, which can allow for the safe and effective use of the radiopharmaceutical in clinical applications. The study provides new insights into the biodistribution and dosimetry of ^89^Zr-oxalate and its potential as a wide-spectrum diagnostic radiopharmaceutical.

## 2. Materials and Methods

The coefficients of transition between organs and blood are essential in analyzing the biological distribution of radiopharmaceuticals. However, these coefficients may not be readily available, particularly in the case of therapeutic radiopharmaceuticals. In such cases, it is necessary to estimate these coefficients from clinical and laboratory studies.

One approach to estimating these coefficients involves assuming that the accumulation and excretion of the radiopharmaceutical in the organ follow an exponential law. A suitable exponential function is then selected, and the transition coefficients from the organ to the blood are estimated. Transit coefficients from blood to organs are assessed using a similar approach, if possible.

In cases where accumulation in the organ is impossible, a linear function is used instead. These estimates can be used to create an input file for programs such as WinAct to calculate the activity fraction in each organ and the nuclear transfer. However, it is essential to note that these estimates may have some degree of uncertainty, and should be validated against experimental data wherever possible.

Overall, this method provides a practical approach to estimating the transition coefficients for diagnostic radiopharmaceuticals where such information is not readily available. By accurately estimating these coefficients, it becomes possible to optimize the use of diagnostic radiopharmaceuticals and improve patient outcomes.

### 2.1. Basic Simulation

The initial results from clinical data on radiopharmaceuticals have often been obtained from various publications. These publications typically describe the biological distribution of the radiopharmaceutical in a mouse or human body, and may include an analysis of the dependence of %ID/g (percentage of injected dose per gram of tissue) on time post-administration.

Such analyses can provide valuable insights into the pharmacokinetics and biodistribution of radiopharmaceuticals, and can help to optimize their use in diagnostic and therapeutic applications. For example, the analysis of %ID/g over time can reveal important information about the clearance of the radiopharmaceutical from various organs and tissues, as well as its accumulation in target tissues such as tumors.

The results from analyses of the biological distribution of radiopharmaceuticals in a mouse or human body are typically presented as a graph, table, or histogram. These visualizations can help to illustrate the dependence of %ID/g on time post-administration, as well as any patterns or trends that may be present.

Table 1 displays the outcomes, initially shown in a tabular format, indicating the source of experimental data employed for the present analysis [8]. To obtain numerical values from the graph, a special program called GetData Graph Digitizer 2.26 Rus Portable was used to digitize the results. This program extracts the data from the image and converts it into a digital format for further analysis. The resulting data represents the percentage of injected dose per kilogram of tissue (%ID/kg) plotted against time after injection. The calculation of %ID/kg is determined by a formula [20]: $\%\frac{ID}{kg}=\frac{Activity\;in\;the\;organ\;(Bq/Kg)}{Input\;activity\;(Bq)}\times 100\%$. This takes into account the amount of radioactivity present in the tissue sample and the weight of the sample. By digitizing the data, it becomes easier to analyze it and compare the results with other studies.

When analyzing the biological distribution of radiopharmaceuticals, it is important to consider the radioactive decay of the radionuclide over time. This decay results in a decrease in the total amount of radioactivity present in the body and can have a significant impact on the accuracy of the results.

To address this issue, the results of biological distribution analyses are often presented in terms of %ID/kg, which takes into account the radioactive decay of the radionuclide. However, in some cases, the results may be presented in MBq/kg, which does not account for the decay.

To convert the results from MBq/kg to %ID/kg, it is necessary to determine the transition coefficients from blood to organs and vice versa. These coefficients can be evaluated using statistical software such as STATISTICA 10 or OriginPro 2021b. The specific activity of the radiopharmaceutical (kBq/g) [8] is typically modeled as a function of time using an exponential decay equation, such as $A\left(t\right)=A\times e^{-at}$.

By applying these techniques, it is possible to accurately analyze the biological distribution of radiopharmaceuticals and account for the effects of radioactive decay over time. This information can be used to optimize the use of radiopharmaceuticals in diagnostic and therapeutic applications, and to improve patient outcomes.

**Figure 1:**
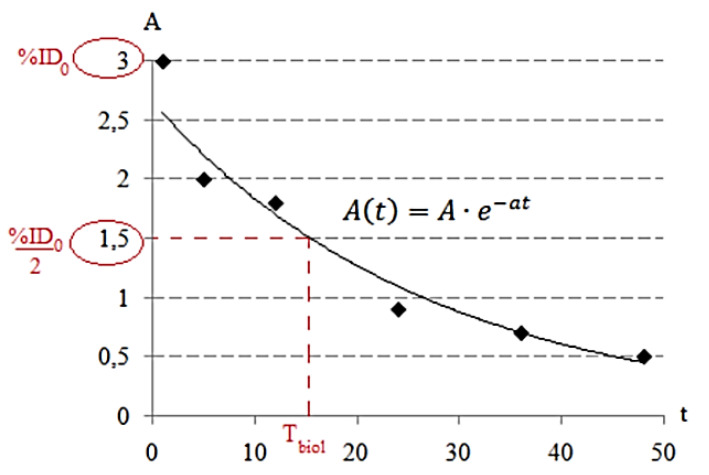
Mathematical model of the dynamics of changes in the concentration of radiopharmaceuticals over time in the organ.

Data on biological half-lives (*T_biol_*) were calculated based on an exponential curve and on the assumption that the activity of a radionuclide in the body tissues decreases over time (Figure 1). If the specific activity A and the rate constant accurately match when constructing the curve, then the point with a value of half of the entered activity will correspond to the biological half-life *T_biol_*. Thus, using the formula $A\left(t\right)=A\times e^{-at}$, it is easy to express the biological half-life of *T_biol_*:

$$T_{biol}=\frac{1}{a}\times \ln\left(\frac{2A}{\%ID_{0}}\right)$$

where *T_biol_* is the biological half-life of the drug from the organ or tissue, h; a is the rate constant, h^−1^; and %ID_0_ is the initial percentage of the injected dose in the organ or tissue, %/organ. Data on effective half-lives were calculated using the formula:

$$T_{eff}=\frac{T_{biol}\times T_{\frac{1}{2}}}{T_{biol}+T_{\frac{1}{2}}}$$

where *T_eff_* is the effective half-life of the drug, h; *T_biol_* is the biological half-life of the drug from an organ or tissue, h; and *T*_1/2_ is the physical half-life of the radionuclide, h.

### 2.2. WinAct Data

To create the input file for the WinAct program, one of the key steps is to calculate the coefficients of transfer for each organ. These coefficients represent the fraction of activity that is transferred from the source region (e.g., blood) to each target organ, and are a critical input parameter for the program’s calculations [21,22,23].

The coefficients of transfer can be estimated using a variety of methods, such as compartmental modeling or fitting of time–activity curves (Figure 1). These methods involve measuring the radioactivity in various regions of the body over time and using mathematical models to estimate the transfer of activity between these regions. For example, in a study involving the use of a radiopharmaceutical for cancer imaging, the coefficients of transfer might be calculated by measuring radioactivity in the blood, liver, spleen, and tumor over time. Mathematical models could then be used to estimate the transfer of activity from the blood to each organ, as well as the accumulation of activity in the tumor.

Once the coefficients of transfer have been calculated, they can be used to create the input file for the WinAct program Supplementary Figure S1. This file typically includes information such as the administered dose, the half-life of the isotope, the patient’s body weight and dimensions, and the coefficients of transfer for each organ. The program then uses this information to calculate the activity distribution to each organ and the nuclear transfer of the radioactive isotope. Overall, the calculation of coefficients of transfer is a critical step in the creation of input files for the WinAct program, requiring careful measurement and modeling of radioactivity in various regions of the body. Accurate estimation of these coefficients is essential for optimizing the design and dosing of radiopharmaceuticals and for ensuring the safety and efficacy of these agents in diagnostic and therapeutic applications.

This study is focused on estimating the coefficients of transition for radiopharmaceuticals. This information can be found in ICRP Publication 128 for diagnostic radiopharmaceuticals, but the list of radiopharmaceuticals is not complete and does not describe new or perspective preparates. To overcome this, the researchers in this study used various clinical and laboratory studies to estimate these coefficients.

**Figure 2:**
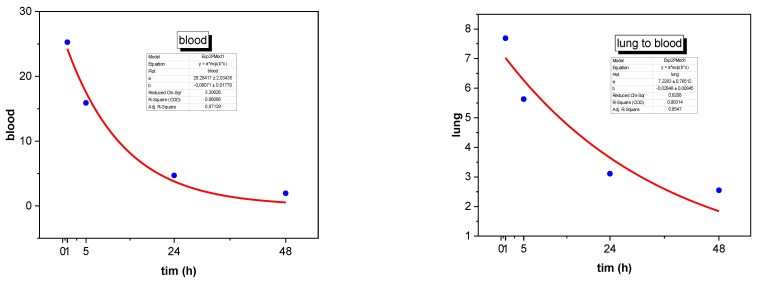

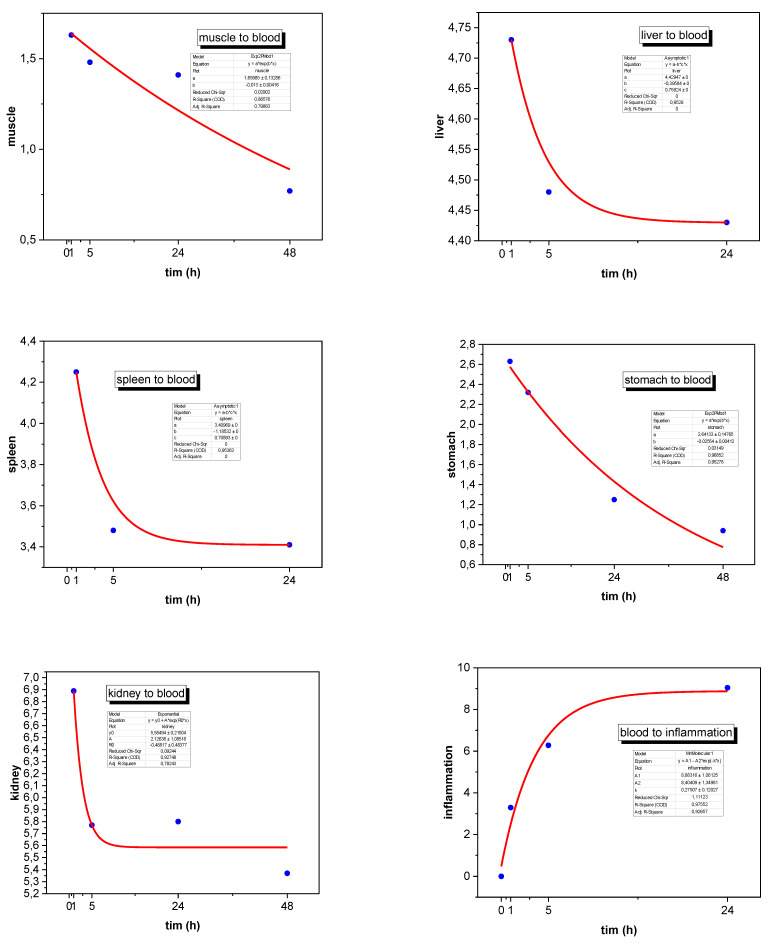

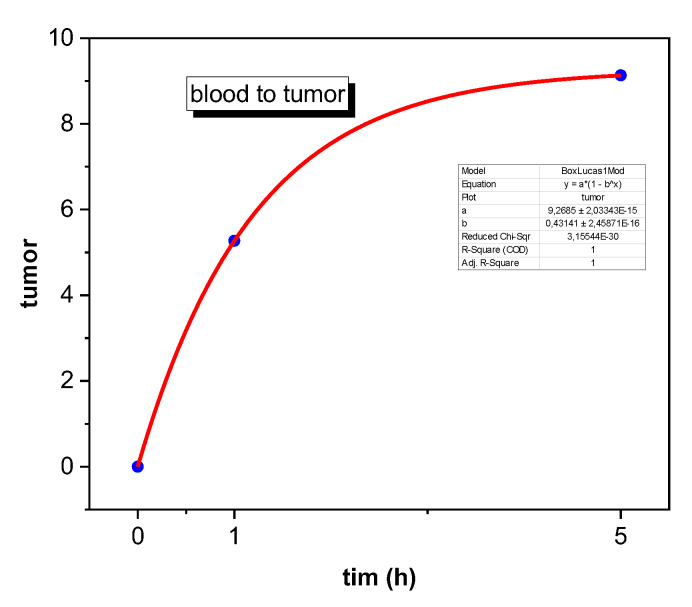
Transfer decay: an exponential function fitting estimated coefficients of transition from the organs to the blood and from the blood to the organs.

An exponential function was selected to describe the accumulation and excretion of the radiopharmaceutical in the organs, which was based on the assumption that these processes follow an exponential law Figure 2. The coefficients of transition from the organs to the blood and from the blood to the organs were estimated using results obtained from the clinical data. These results were presented in the form of tables, graphs, or histograms, and, if necessary, digitized to obtain numerical values. The coefficients were evaluated using the STATISTICA 10 and OriginPro 2021b programs see Figure 2.

The parameters for the exponential function were evaluated based on the results from Figure 1, which show the dependence of %ID/kg on time after administration. The coefficients were calculated as the parameters for the biokinetic model of the radiopharmaceutical. In cases where the accumulation of activity in the organ was impossible, a linear function was considered to simplify the evaluation. The results of the evaluation [8] are presented in Table 2.

**Figure 3:**
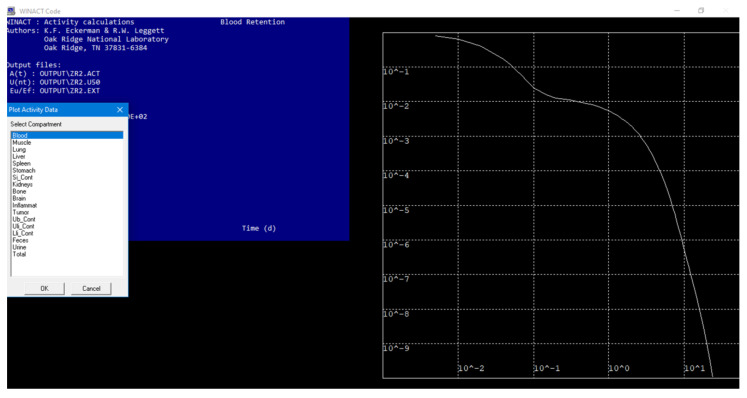
WinAct Running.

The distribution of radiopharmaceuticals in the human body can be described using a system of differential equations of the first order. However, solving such a system can be extremely complex, and the number of equations can reach 20 to 40. To overcome this challenge, WinAct software can quickly solve the system of equations [22]. The main parameters required for calculating the radiopharmaceutical distribution are the transition coefficients (day^−1^) from the blood to the organs and vice versa, represented in the diagrams shown in Figure 3.

When the input file is created, WinAct generates three output files and an information file with a “.log” extension that duplicates the input data. The file with the “.act” extension contains information about the activity present in an organ or tissue as a function of time since the administration of the radionuclide. This file is used to plot the retention of activity in relation to time after administration. The file with the “.ext” extension contains data regarding the rate of excretion of the radionuclide with urine and feces (1/day) as a function of time, as well as tabulated data on the retention of the radionuclide in the lungs and body as a function of time.

The “.u50” file contains data on the number of nuclear transformations in a particular organ or tissue. The output results from the WinAct program are used as input data for the IDAC-2.1-software, an internal dosimetry program for nuclear medicine based on ICRP adult reference voxel phantoms [24,25]. This allows for the calculation of absorbed doses in different organs and tissues. One of the WinAct output files provides information about the amount of decay in the source organ, which is used to calculate the resident time or cumulative activity for the source organs. The resident time is used to calculate the dose coefficients (mGy/MBq) using IDAC-Dose 2.1 [24,26].

To obtain the number of nuclear transformations per Bq intake as a function of time (d), the number of nuclear transformations obtained from WinAct is divided by the mass of the mouse organ [27], as listed in Table 3. Since most ^89^Zr-oxalate are tested in mice with implanted tumors (left shoulder) and inflammation (right thigh), it is necessary to convert the cumulative activity in mice to that in humans (Table 4) using the Sparks and Aydogan method, as described in the following Equation [28].

$${\tilde{A}}_{human\;organ}={\tilde{A}}_{animal\;organ}\left[\frac{{\left(\frac{organ\;mass}{body\;mass}\right)}_{human}}{{\left(\frac{organ\;mass}{body\;mass}\right)}_{animal}}\right]$$

The calculation of the absorbed dose in organs and tissues was accomplished by utilizing two methods. The first approach involved the use of the WinAct program to estimate the transfer activity fraction from the blood to organs, the removal activity rate from the organs, and the excretion rate [22]. WinAct software uses a system of differential equations of the first order to describe the radiopharmaceutical distribution in the human body, which can be a complex task if solved manually. However, with the help of WinAct software, solving this system becomes much more manageable.

In the second approach, the output from the WinAct program was inserted as input data into IDAC-2.1-software. This software is an internal dosimetry program for nuclear medicine that is based on ICRP adult reference voxel phantoms [25,29]. The program uses cumulated activity data in organs and tissues to estimate the absorbed doses in each organ or tissue.

It is important to note that most ^89^Zr-oxalate radiopharmaceuticals are tested in mice, and, therefore, it is necessary to convert the cumulated activity in mice to that in humans. This can be accomplished using the Sparks and Aydogan method [28]. The final output from the IDAC-2.1-software provides an estimate of the absorbed doses in each organ or tissue of the human body.

## 3. Results and Discussion

**Figure 4:**
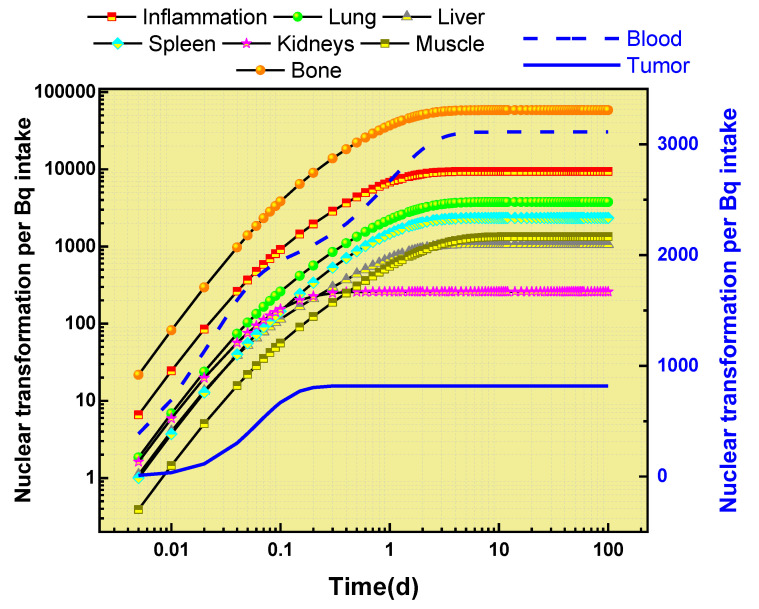
Nuclear transformation per Bq intake of ^89^Zr-oxalate with time (d) for some organs.

The results of the analysis of ^89^Zr-oxalate’s behavior in different organs are presented in Figure 4. This figure shows the maximum amount of nuclear transformation per Bq intake for various organs, such as the bones, blood, muscle, liver, lung, spleen, kidneys, inflammation, and tumors. These results were obtained using the WinAct software program, and are part of the output files generated by the program.

The analysis showed that ^89^Zr-oxalate has the greatest impact on the bone, causing the highest level of nuclear transformation in this organ. On the other hand, it has a relatively minor impact on the kidneys and tumors, with lower levels of nuclear transformation compared to other organs.

In addition to the amount of nuclear transformation, Figure 4 also provides information about the retention time of the maximum nuclear transformation in each organ. This information provides a comprehensive view of ^89^Zr-oxalate’s behavior in different parts of the human body.

**Figure 5:**
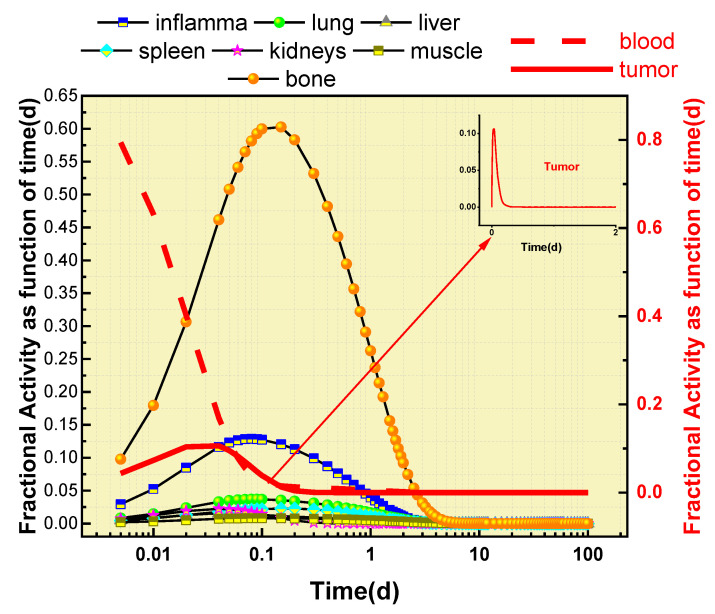
Activity fraction of ^89^Zr-oxalate as a function of time for some organs.

The results in Figure 5 illustrate the retention pattern of ^89^Zr-oxalate in the blood and various organs, including bones, inflammation, lungs, liver, kidneys, spleen, and tumors. The aim is to minimize the absorbed dose in human organs and tissues, so ^89^Zr-oxalate should quickly leave the bloodstream once its intended effect on the area with increased metabolism is achieved. In the presented results, the bones are shown to have the highest fraction of activity, followed by inflammation, compared to the other organs. The time–activity fraction information for each organ was used as input data in the IDAC-2.1-software to estimate the absorbed doses in various organs and tissues. The figure displays the maximum fraction activity in the main organs and the time of intake for ^89^Zr-oxalate. Notably, the time of intake for the bone is six times higher than for the other organs.

**Figure 6:**
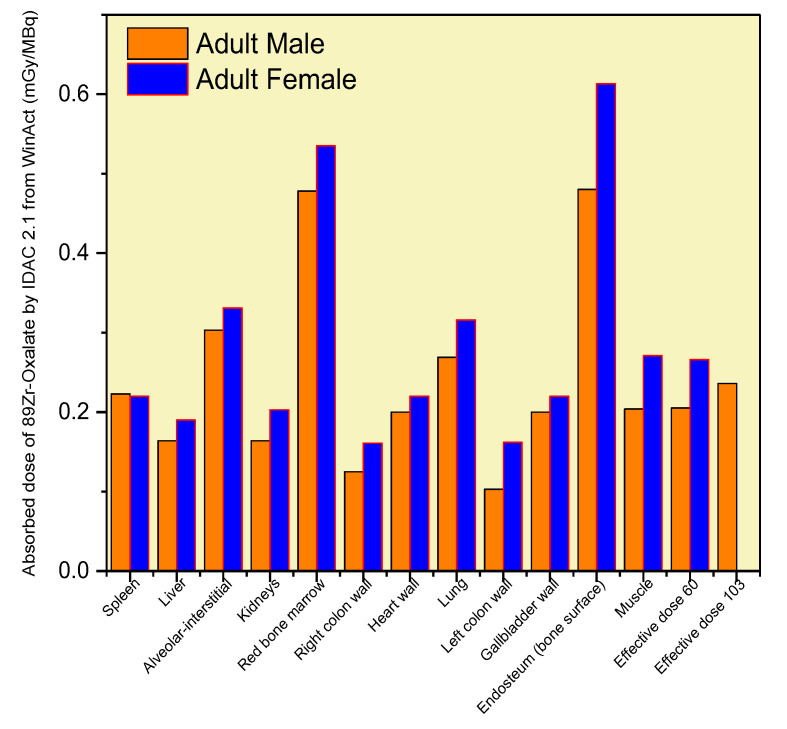
^89^Zr-oxalate absorbed dose in mGy/MBq over time for certain organs, calculated by the IDAC-2.1-software using input data from the WinAct program.

Figure 6 presents the absorbed doses in mGy/MBq for ^89^Zr-oxalate in various organs and tissues. The results show that the absorbed dose in the bone surface was three times higher than in the liver, and the red bone marrow received the highest dose among all organs. On the other hand, the left and right colon received the lowest absorbed doses compared to other organs. Table 5 provides a detailed overview of the absorbed doses for the alveoli, bronchioles, bone surface, heart wall, kidneys, liver, lungs, lymphatic nodes, and spleen, indicating a wide range of absorbed doses across the various organs and tissues.

For tumors and inflammations, the absorbed dose was calculated separately for each unit volume sphere using the information on the injected dose. The results indicate that ^89^Zr-oxalate has a favorable biodistribution, with high bone uptake and little effect on healthy organs.

Overall, the results show that the absorbed doses of ^89^Zr-oxalate in various organs and tissues depend on the specific biodistribution properties of this radiopharmaceutical. The favorable biodistribution properties of ^89^Zr-oxalate make it a promising candidate for wide-spectrum diagnostic radiopharmaceuticals. These findings demonstrate the importance of assessing the absorbed doses of radiopharmaceuticals to ensure their safe and effective use in clinical applications.

This study on ^89^Zr-oxalate has provided useful information on the drug’s biodistribution on bone metastasis. The results indicate that the drug has a high affinity for bones, as evidenced by substantial nuclear transformation in this tissue. Furthermore, the drug appears to have a relatively low impact on healthy organs, such as the kidneys, compared to other organs.

Figure 4 and Figure 5 present important information on the retention time and activity fractions of ^89^Zr-oxalate in various organs and tissues. The maximum nuclear transformation per Bq intake for the main organs is also presented in Figure 4. The absorbed doses in mGy/MBq for ^89^Zr-oxalate in various organs and tissues are shown in Figure 6. It can be observed that the absorbed dose in the bone surface was three times higher than that in the liver, while the red bone marrow received the highest absorbed dose compared to other organs. The left and right colon received the lowest absorbed dose compared to other organs.

The results suggest that ^89^Zr-oxalate has a high affinity for bone, as evidenced by the substantial nuclear transformation observed in this tissue. The study found that the absorbed dose in the bone surface was three times higher than that in the liver, and the red bone marrow received the highest dose among all organs. The study provides valuable information for further research on the development of this drug for potential clinical applications.

## 4. Conclusions

The results of this study on ^89^Zr-oxalate suggest that it has a very favorable biodistribution, with high bone uptake and low impact on other healthy organs. The maximum nuclear transformation was observed in the bones, while minor transformation was observed in the kidneys and tumors. ^89^Zr-oxalate had the highest activity fraction in the bones compared to other organs, and the time of intake was six times higher for the bones than for other organs. The time of maximum transformation for each organ shows the dependence of ^89^Zr-oxalate retention on the blood and main organs (bones, inflammation, lungs, liver, kidneys, lung, spleen, tumors). The absorbed dose in the bone surface was three times higher than that in the liver, and the red bone marrow received a much higher dose compared to the other organs. The absorbed doses in tumors and inflammations were calculated separately, and were found to be low. Based on these results, it can be concluded that ^89^Zr-oxalate may be a suitable candidate for wide-spectrum diagnostics due to its high bone uptake and low effect on healthy organs. Further studies are needed to fully assess the safety and efficacy of ^89^Zr-oxalate.

## Figures and Tables

**Table 1:** Biodistribution data (%ID/g) of ^89^Zr-oxalate in the tumor and inflammation models.

Tissue	1-h	5-h	24-h	48-h
Blood	25.27 ± 3.01	15.90 ± 1.44	4.71 ± 0.53	1.95 ± 0.30
Muscle	1.63 ± 0.35	1.48 ± 0.41	1.41 ± 0.73	0.77 ± 0.30
Lung	7.69 ± 1.33	5.63 ± 1.25	3.11 ± 0.39	2.55 ± 0.30
Liver	4.73 ± 0.74	4.48 ± 0.45	4.43 ± 0.40	4.73 ± 0.98
Spleen	4.25 ± 0.59	3.48 ± 0.82	3.41 ± 0.70	4.07 ± 0.85
Stomach	2.63 ± 0.85	2.32 ± 0.53	1.25 ± 0.35	0.94 ± 0.26
Intestine	2.73 ± 0.41	2.81 ± 0.33	1.18 ± 0.22	1.00 ± 0.22
Kidney	6.89 ± 1.22	5.77 ± 0.73	5.80 ± 0.81	5.37 ± 0.95
Bone	6.38 ± 1.89	11.76 ± 4.31	20.67 ± 6.35	28.63 ± 9.63
Brain	0.79 ± 0.18	0.37 ± 0.08	0.21 ± 0.06	0.14 ± 0.02
Inflammation	3.29 ± 0.94	6.28 ± 1.34	9.04 ± 2.68	7.96 ± 0.94
Tumor	5.27 ± 1.16	9.13 ± 1.85	7.45 ± 0.81	6.22 ± 0.73
Inflammation/Blood	0.13 ± 0.03	0.39 ± 0.06	1.94 ± 0.64	4.09 ± 0.27
Inflammation/Muscle	2.19 ± 1.17	4.50 ± 1.59	6.90 ± 1.57	11.14 ± 3.07
Tumor/Blood	0.21 ± 0.06	0.57 ± 0.08	1.59 ± 0.25	3.20 ± 0.24
Tumor/Muscle	3.44 ± 1.41	6.36 ± 1.22	5.99 ± 1.87	8.68 ± 2.27

**Table 2:** Coefficients of transfer, 1/day, for ^89^Zr-oxalate (mice with tumors).

Organ	Organ to Blood	Blood to Organ
Blood	0.000	1.937
Muscle	0.312	0.391
Lung	0.684	1.846
Liver	18.222	1.135
Spleen	17.014	1.020
Stomach	0.613	0.631
Intestine	0.579	0.655
Kidney	11.716	1.654
Bone		21.540
Brain	1.451	0.190
Inflammation		6.602
Tumor	0.000	10.354

**Table 3:** Organ mass of mice and humans and the coefficient factor from mouse to human.

	Organ Mass in Humans (g)	Organ Mass in Mice (g)	Coefficient from Mouse to Human
Organs	Male	Male	Male
Liver	2360	2.01	0.45
Kidney	422	0.48	0.34
Spleen	228	0.13	0.66
Muscles	29,784	17.12	0.67
Brains	1517	0.44	1.32
Bone	10,000	1.82	2.11
Lungs	1200	0.10	4.81
Stomach	150	0.17	0.34
Small intestine	640	0.88	0.28
Urinary bladder	51.10	0.14	0.14
Blood	5500	2.45	0.86
Tumor	62.4002	0.024	1.00
Inflammation	62.4002	0.024	1.00
Total weight	70,000	26.92	1.00

**Table 4:** Conversion of cumulated activity from mice to humans.

	Mice	Human
Time (h)	2400	
Blood	7627.13	6581.577
Muscle	23,017.23	15,404.01
Lung	361.6992	1738.933
Liver	2138.64	965.7819
Spleen	309.5136	205.6203
Stomach	240.1464	0.34
si_cont	157.7151	44.21649
Kidneys	123.5917	41.96616
Bone	106,253.4	224,541.7
Brain	93.24432	123.0867
Inflammation	225.7128	225.7128
Tumor	19.64088	19.64088
ub_cont	742.4852	106.5157

**Table 5:** Estimation of absorbed dose of ^89^Zr-oxalate in various organs using IDAC 2.1 and WinAct, measured in mGy/MBq.

Organs (mGy/MBq)	Adult Male	Adult Female
Adrenals	0.211	0.325
Alveolar–interstitial	0.303	0.331
Brain	0.314	0.356
Breast	0.074	0.1
Colon wall	0.132	0.166
Endosteum or bone surface	0.48	0.613
Gallbladder wall	0.124	0.226
Heart wall	0.2	0.22
Kidneys	0.164	0.203
Left colon wall	0.103	0.162
Liver	0.164	0.19
Lung	0.269	0.316
Lymphatic nodes	0.213	0.243
Muscle	0.204	0.271
Esophagus	0.323	0.379
Oral mucosa	0.338	0.483
Ovaries	0	0.233
Pancreas	0.149	0.178
Prostate	0.211	0
Recto-sigmoid colon wall	0.206	0.186
Red bone marrow	0.478	0.535
Right-colon wall	0.125	0.161
Skin	0.117	0.134
Small intestine wall	0.15	0.172
Spleen	0.223	0.22
Stomach wall	0.127	0.146
Testes	0.082	0
Thymus	0.272	0.269
Thyroid	0.261	0.284
Ureters	0.2	0.276
Urinary bladder wall	0.21	0.224
Uterus/cervix	0	0.209
Effective dose 60 (mSv/MBq)	0.205	0.266
Effective dose 103 (mSv/MBq)	0.236	

## Data Availability

Available with request.

## Supplementary Materials

The following supporting information can be downloaded at: https://www.mdpi.com/article/10.3390/biomedicines11041173/s1, Figure S1: WinAct input file of ^89^Zr-oxalate.
