# Supplementary material for: Exploring the Potential of Zirconium-89 in Diagnostic Radiopharmaceutical Applications: An Analytical Investigation
Source: Biomedicines. 2023 Apr 13;11(4):1173. doi: 10.3390/biomedicines11041173 (PMC10135474; doi:10.3390/biomedicines11041173)
Supplement: Supplementary file 1 [file biomedicines-11-01173-s001.zip › biomedicines-2280832-supplementary.pdf]

```
This file is Zr.INP.  This case is an acute injection of Zr-89
THALF 3,27E+00                              <- Halflife (d)
COMPARTMENTS  A(0)                          <- Delimiter for initial condition block
Blood_1    1.0
END LIST                                    <- Delimiter for end of block
TRANSFERS              (/d)                 <- Delimiter for start of transfer data
Blood_1      ->Muscle    0.391
Blood_1      ->Lung      1.846
Blood_1      ->Liver     1.135
Blood_1      ->Spleen    1.020
Blood_1      ->Stomach   0.631
Blood_1      ->SI_Cont   0.655
Blood_1      ->Kidneys   1.654
Blood_1      ->Bone      21.540
Blood_1      ->Brain     0.190
Blood_1      ->Inflammat  6.602
Blood_1      ->Tumor     10.354
Blood_2   ->UB_Cont              53.3
Muscle      ->Blood_2     0.312
Lung        ->Blood_2     0.684
Liver       ->Blood_2     18.222
Spleen      ->Blood_2     17.014
Stomach     ->Blood_2     0.613
SI_Cont     ->Blood_2     0.579
Kidneys     ->Blood_2     11.716
Brain       ->Blood_2     1.451
Bone        ->Liver      0.300
Bone        ->Spleen     0.660
Inflammat   ->Bone       1.080
Tumor       ->Bone       23.398
Blood_1   ->ULI_Cont             1.29E-02
SI_Cont   ->ULI_Cont             6.00E+00
ULI_Cont  ->LLI_Cont             1.80E+00
LLI_Cont  ->Feces              1.00E+00
UB_Cont   ->Urine              12.0E+00
EOF Data
```

Figure S1. WinAct input file of ^{89}Zr-oxalate
